# Optimization Potential of Ecosystem Functions of Tree and Shrub Plantations in Anthropogenically Transformed Territories of the Southern East European Plain

**DOI:** 10.3390/biology15100784

**Published:** 2026-05-14

**Authors:** Vladimir Kornienko, Inna Pirko, Besarion Meskhi, Anastasiya Olshevskaya, Mary Odabashyan, Arkady Mirzoyan, Sergey Zolotov, Denis Kozyrev

**Affiliations:** 1 Scientific Research Laboratory for Monitoring and Forecasting of Donbass Ecosystems, Donetsk State University, 24 Universitetskaya St., Donetsk 283001, Russia; pirko.i.f@mail.ru; 2 Agribusiness Faculty, Don State Technical University, Rostov-on-Don 344000, Russia

**Keywords:** anthropogenic transformation, green infrastructure, ecological corridors, native tree and shrub communities, resource potential

## Abstract

The anthropogenic transformation of the steppe zone in the southern East European Plain has led to the destruction and catastrophic fragmentation of natural ecosystems. Due to the presence of highly fertile lands and the deposits of the Donetsk coal basin, up to 90% of the territory is occupied by agricultural and industrial activities, urban agglomerations, other settlements, and extensive transportation networks. The predominant use of introduced species in artificial plantings (within the city limits, the ratio of species to quantity is 7:3) leads to the widespread spread of alien species, further isolation of natural habitats, and their subsequent degradation. The problem of preserving natural ecosystems and restoring a stable balance in their functioning can be solved through the widespread introduction of native species into all types of plantings capable of serving as ecological corridors. In this regard, we analyzed the key characteristics of native tree and shrub species that determine their functional value. The results indicate that of the 85 native plant species, only two cannot be used because they carry pests and diseases dangerous to agricultural crops. The remaining 83 species are suitable for various planting types, based on a set of individual characteristics, and 29 of these are universal for all planting types. Outside urban ecosystems, these 83 native species can completely replace introduced species. Within urban ecosystems, the need for their combination remains. Despite a number of advantages identified in native species in conditions of anthropogenic pollution (relatively high viability, long lifespan, good resistance to mechanical stress), native species lack a number of categories of traits necessary for the more effective functioning of urban green infrastructure. Among them, there is an insufficient number of tall species (>25 m) and conifers, which are more effective in purifying and improving the health of the atmosphere, as well as beautifully flowering and generally highly decorative species necessary for recreational areas and other territories that, among other things, perform esthetic functions.

## Simple Summary

This article addresses current issues of restoration and stabilization of the regional ecosystem in the steppe zone of the southern East European Plain through the reconstruction and expansion of tree and shrub plantation networks. These plantations serve as fundamental elements of the green infrastructure of urban ecosystems and the ecological framework of the territory, yet currently exist in a state of digression under progressive anthropogenic transformation. Within the concept of ecological framework formation and territorial ecological stability, minimization or complete replacement of introduced species with native ones is recommended, which is particularly important for green zones functioning as ecological corridors between framework elements. A comprehensive analysis of the native fraction of the regional tree and shrub communities demonstrates its adaptive and functional potential, enabling the effective performance of environment-forming, protective, and nature conservation functions.

## 1. Introduction

The environmental consequences caused by anthropogenic activities are of serious concern: environmental pollution, depletion of natural resources, reduction in biodiversity, biological invasions, etc. However, the most serious threat is the loss and/or significant fragmentation of natural habitats as a result of the intensive expansion of agricultural, urban and anthropogenic landscapes [1,2,3,4,5,6,7,8,9,10,11,12]. These processes significantly reduce or eliminate the possibility of self-healing of natural ecosystems. This is especially relevant for densely populated areas, which include the steppe zone of the southern East European Plain. The study area covers 2651.7 thousand hectares, of which 100.2 thousand hectares are areas devoid of vegetation (sand, pebbles, scree, landslides, ravines, etc.), and 42.8 thousand hectares are areas covered by surface water [13]. Agricultural lands make up 2041.1 thousand hectares, and built-up areas—200.3 thousand hectares. Thus, anthropogenically transformed lands occupy 89% of terrestrial ecosystems. Such large-scale fragmentation of habitats and the active spread of invasive species across anthropogenically modified lands are the most significant factors in the reduction in biodiversity [8,14]. As a result, all these interconnected processes lead to an imbalance in the ecosystem [14,15].

Currently, military activity is the dominant factor in the pollution, loss, and fragmentation of natural ecosystems and artificial plantings in the study area [16,17,18]. Its consequences, combined with the lack of planned conservation measures over the past three decades, have led to the development of an ecological disaster [10,11,12]. Trees and shrubs, being the most vulnerable and difficult to restore component of steppe ecosystems, are at greatest risk. Military actions, numerous illegal loggings, and natural depletion due to harsh climatic conditions and reaching a critical age [12,19] lead to its complete destruction or significant damage.

Addressing this problem requires optimization of natural resource management strategies and modern approaches to restoring the ecological framework of the territory (EFT). The EFT represents a system of natural and cultural landscapes, environment-forming and environment-regulating biomes, and elements possessing the greatest ecological resilience, capable of ensuring territorial ecological stability and maintaining a sustainable natural balance of matter and energy, landscape and species diversity [20,21,22,23,24,25]. An essential component of the EFT includes not only the natural framework—i.e., the aggregate of undisturbed interconnected territories—but also semi-natural territories that may serve as supplementary corridors facilitating interactions between natural ecosystems [22,23,26]. Such entities, potentially suitable for performing ecological functions following restoration interventions, may include the green infrastructure of urban ecosystems, forest amelioration, water-protection, field-protection, roadside, and other artificial plantations, provided that introduced species are minimized or completely replaced therein.

The dominance of alien species in green plantations, despite certain esthetic and functional advantages attributable to the florogenetic characteristics of the steppe zone, is associated with a range of negative consequences for regional ecosystems, including disruption of ecological linkages, invasion of natural phytocoenoses, displacement of native species, threats to human health, and high resource intensity [3]. The use of native species in the extensive network of shelterbelts on agricultural land, in roadside plantings along the well-developed road network of this densely populated area, and in other types of tree and shrub plantings would help restore and optimize natural biological flows between the main elements of the ecological structure. This approach could contribute to the restoration and maintenance of self-regulation and self-preservation processes in ecosystems at various hierarchical levels, from local to regional.

Thus, the aim of this study is to determine the resource potential of native tree and shrub communities in the southern East European Plain for the formation of highly effective green infrastructure in urbanized and other anthropogenically transformed areas. To achieve this, two objectives were set: (1) the analysis of key characteristics determining the adaptive and functional value of native species, and (2) the identification of the most optimal options for species use.

## 2. Study Area

**Figure 1:**
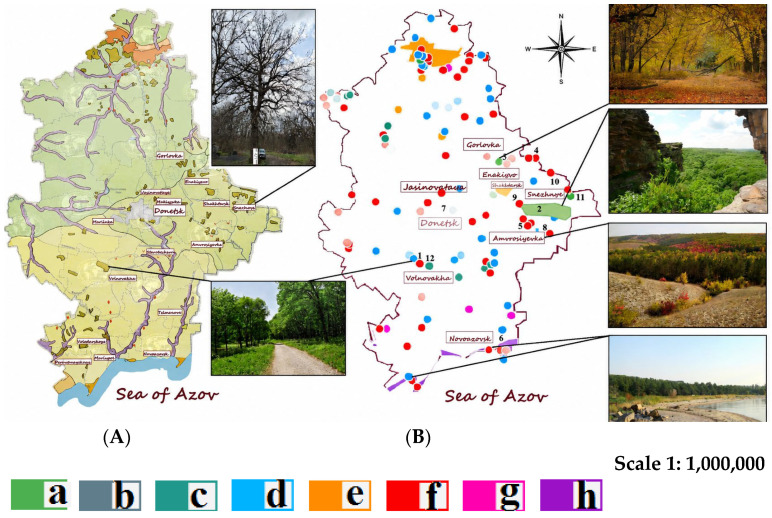
The territory of field research of the native flora of the South of the East European Plain. Notes: Vegetation map (**A**) and Nature Reserve Fund (**B**) of the Donetsk region (based on the ecological atlas of the Donetsk region [13]). Studied territories: 1. Velikoanadolsky Forest (Volnovakha district); 2. Donetsk Ridge Regional Landscape Park; 3. Rossokhovatoye Tract Forest Reserve, Yenakiyevo Forestry; 4. Skelevaya Balka Landscape Reserve (Yenakiyevo City); 5. Berdyansky Forest Reserve, Krynka River Forest Landscape Reserve (Amvrosiyevsky district); 6. Khomutovskaya Steppe—Meotida Biosphere Reserve, Novoazovsky district; 7. Donetsk Botanical Garden (Donetsk) and Donetsk Urban Forest Ecosystems; 8. Avrosiyevsky Chalk Isolate Specially Protected Natural Area (Amvrosiyevsky district); 9. “Obushok” Botanical Reserve (Shakhtyorsky district); 10. Local Forest Reserve (Snezhnoye and its environs); 11. “Gornyatsky Oak, Mius ravine forests of the Donetsk Ridge” Botanical Natural Monument. 12. Mariupol Forest Dacha Reserve. Map legend: (a) Parks-monuments of landscape gardening art; (b) Botanical Garden; (c) Protected areas; (d) Natural monuments; (e) Landscape parks; (f) State nature reserves; (g) Nature reserve branches; (h) Biosphere specially protected natural territory of republican significance “Khomutovskaya steppe—Meotida”; Amvrosievsky chalk isolate.

The study area is situated within the southern steppe subzone of the East European Plain, encompassing the western portion of the Donetsk Ridge and the eastern part of the Azov Upland, which transitions into a narrow strip of the Azov Lowland descending toward the Azov Sea and currently almost entirely under cultivation (Figure 1). The territory contains the watershed of 247 predominantly low-water rivers, the largest being the Seversky Donets—a major tributary of the Don River. Industrial effluents supplement all rivers, in addition to natural recharge. The terrain is undulating, characterized by severe soil erosion. The typical landscape consists of heavily dissected plains and uplands intersected by gullies (balkas), transitioning into floodplain landscapes of river valleys, as well as liman plains along the seacoast. The dominant vegetation type is steppe; forest phytocoenoses are represented insignificantly, predominantly by artificial plantations [10,27].

The climate is moderately continental and arid. Average annual precipitation ranges from 450 to 550 mm. There are 90–120 days with precipitation per year. The winter months receive 20–35% of the annual precipitation, which serves as the main reservoir of moisture accumulated in the soil. Snow cover is generally shallow and unstable at 3–9 cm. About half of the annual precipitation falls in the summer, but only a small portion of it infiltrates the soil and is used by plants, as the precipitation falls as torrential rains. In dry years, precipitation in the coastal areas of the Sea of Azov can drop to 100 mm. Overall, the region is characterized by a moisture deficit (humidification coefficient of 0.6–0.5). Prevailing winds during cold weather are easterly and northeasterly, with maximum speeds of 15–20 m/s. Windbreaks and windthrows are common causes of damage to trees. This is especially common in winter, combined with icing or wet snow accumulation. Dust storms and dry winds are common in the southern and southeastern regions, especially in May. Relative humidity during this period can drop to 11–14%.

The annual total radiation in the region is 105–115 kcal/cm^2^. The lowest radiation influx is observed in winter, with a minimum in December; in summer, the temperature rises sharply, reaching a maximum in July. The average annual temperature fluctuates from 7.1 °C in the northeast to 8.6 °C in the south. The average temperature of the coldest month (January) fluctuates between −6.0 and −7.8 °C. A characteristic feature of the climate is frequent thaws in winter and the associated icing. The maximum air temperature recorded in winter is +15 °C. The absolute minimum is −35 °C. In the warmest month (July), the average temperature is 20.9–24.0 °C. The absolute maximum is 40 °C. The frost-free period lasts 170–210 days. However, the growing season is often shortened due to late spring (end of May) and early autumn (beginning of September) frosts [13,28].

The soils are chernozem, formed under the insufficiently moistened conditions of the northern steppe zone on smoothed loess-like loams. The humus horizon in the soil profile is 60–80 cm thick, with a humus content of 5–8%. It has a humate character, is rich in calcium, and is well fixed in the soil. In the northeastern part, there are islands of chernozem on solid carbonate deposits. On the Azov Upland, chernozems on the weathering products of hard rock are common. These soils have a brown tint and lower fertility compared to typical and ordinary chernozems. The soil cover of the Azov Lowland consists of ordinary (medium-humus) chernozems; southern (low-humus) chernozems, dark chestnut, and solonetzic soils are less common. In the valley of the Seversky Donets River, gleyed soils are found, on the Azov spits (Belosaraiskaya and Krivaya) and along the banks of the Seversky Donets—sands and sandy loams, in the valleys of some rivers—swamp–meadow soils.

In addition to the difficult natural and climatic conditions, the study area is characterized by an increased level of anthropogenic pollution. Within its limits, there are more than 1500 coal mine dumps containing about 1400 million m^3^ of mining material. These dumps occupy more than 12 thousand hectares of the most fertile soils in the world—chernozems represent zones of primary, most harmful anthropogenic pollution. Fragments of landfills, from blocks to clay particles, are stored mainly in the form of dumps up to 80 m high. The incineration of landfills releases carbon monoxide (CO), nitrogen oxides (NO_x_), sulfur dioxide (SO_2_), coal dust particles and heavy metals. Annual emissions from landfills amount to ~70,000 tons, including CO (38,000 tons), particulate matter (>14,000 tons) and NO_x_ (>5000 tons). The overall level of industrial air pollution in the territory is quite high, despite the socio-economic downturn over the past 10 years, which has led to a reduction in harmful emissions from industrial and transport facilities. Exceedances of the maximum permissible concentrations (MPC) include dust (1.4 times), SO_2_ (2 times), CO (3 times), NO_2_ (2.5 times), NH_3_ (5.5 times), phenol (10 times) and formaldehyde (6.6 times). For soils in the zone of influence of major highways, concentrations of heavy metals exceed the maximum permissible concentration by an average of 40%. The sound pressure levels on Ilyich Avenue exceed the permissible limits by 45–51%, while the frequency spectrum with maximum energy is 400–800 Hz [5].

## 3. Materials and Methods

To achieve these goals, the taxonomic composition of trees and shrubs of the local flora was determined using literary sources. Direct and indirect key characteristics of species that determine their adaptive and functional value are considered: ecomorphological and chorological structure, zonal distribution, cenotic confinement, phytocenotic relationships, as well as some morphological features. The main source of information was a database compiled by the authors based on a study of tree and shrub vegetation in specially protected natural areas (see Figure 1) and plantings in the green infrastructure of the Donetsk–Makiivka agglomeration. Morphobiological and bioecological characteristics are supplemented by data from various scientific sources [19,27,29,30,31,32,33,34,35]. Morphometric and some chronological data are combined into groups based on existing classifications and considered as attributive features. Diagrams were used to visualize the structure of the considered set of species according to one or another feature. When structuring species by flowering and fruiting phenophases, we used time intervals within which these phases vary from year to year (depending on weather conditions). We also took into account the phenophases of the beginning and end of flowering, as well as the phases of fruit ripening and fruit fall.

Height groups were distinguished according to the S.Ya. Sokolov scale [35]: trees—first magnitude (T1, >25 m), second (T2, 15–25 m), third (T3, 10–15 m), and fourth (T4, <10 m); shrubs—first magnitude (S1, >3 m), second (S2, 2–3 m), third (S3, 1–2 m), and fourth (S4, <1 m). Growth rate groups follow A.I. Kolesnikov [33]: fast-growing (Fg, mean annual increment ≥1 m), moderate-growing (Mg, 0.5–0.6 m), and slow-growing (Sg, ≤0.25–0.3 m). Ecomorphs are presented after A.L. Belgard [36,37,38]. Data on natural plant communities containing species of the native fraction of the regional tree and shrub communities are based on phytosociological studies [39,40]. The classification of geo-elements follows the botanical–geographical regionalization scheme of A.L. Takhtajan [41] and other phytogeographical works [1,32,42,43]. Chorological group names reflect the association of species ranges with specific territories and floristic regions.

For verification of taxon nomenclature and natural species ranges, data from the international databases Plants of the World Online [44] and World Flora Online [45] were utilized.

To determine the potential for using native species in the green infrastructure of industrial cities, a comparative analysis of the state of native and introduced species in roadside plantings within the Donetsk–Makiivka agglomeration was conducted.

The condition of trees and shrubs in urban ecosystems and the response of individual species to adverse anthropogenic factors were assessed using an inventory and analysis of plant viability in the stands from 2014 to the present. The survey covered a 13.3 km section of the road network in the Donetsk–Makiivka agglomeration. This section is part of a regional public road (Donetsk–Makiivka–Torez) of category II with an estimated traffic intensity of 3000 to 14,000 traffic units (MU) (with a passenger transport share of ≥30%). Certain morphometric data, morphological characteristics, and plant age were taken into account, which served as the basis for determining the level of individual viability. Shrub height was determined using a tape measure (at least 5 measurements per tract), and branch base diameter (at least 5 branches) was measured using calipers. An HEC Haglof electronic altimeter (Haglöf Sweden AB, Långsele, Sweden, 2017) was used to estimate tree height, and a Haglof Mantax Black measuring caliper (Haglöf Sweden AB, Långsele, Sweden, 2024) was used to measure trunk diameter at breast height (1.3 m) and the base of scaffold branches. Visual inspection data for the studied trees was documented using a Nikon Coolpix S2600 camera (Nikon, Sendai, Japan, 2009). Subsequent office processing and image analysis were performed using Axio Vision Rel. 4.8 software with reference scaling. For all trees, an additional crown inspection was performed (damage area, canopy density, presence of light windows) using photographic recording and subsequent digital processing using Axio Vision Rel. 4.8 software. The area of crown damage was determined using software functions based on the ratio of the total crown area to the area of damage (e.g., areas with chlorotic leaves, leaves damaged by pests, etc.). Plant age was determined based on archival documents from municipal services and the Donetsk Botanical Garden, documenting the planting date and origin of the planting material, archival photographs reflecting the growing conditions of specific specimens, and a number of direct and indirect methods: core sampling (using a Pressler auger) and counting tree rings in cross-sections of thick (skeletal) branches. Because the age structure of the plants in the sample varied greatly (trees ranged in age from 8 to 65 years), for ease of analysis, they were grouped into age classes with 10-year intervals for each tree. The viability of trees and shrubs was assessed based on data obtained in 2023–2024. Tree viability was assessed according to the V.A. Alekseev scale [46]:

− Level 1 (healthy tree)—No external damage to the crown or trunk, crown density typical of dominant species, dead and dying branches concentrated in the lower part of the crown and absent from the upper part, leaves are green or dark green, their lifespan is typical for the region, leaf damage is minor (<10%) and does not affect the condition of the tree.
− Level 2 (damaged (weakened) tree)—At least one of the following characteristics is required: a 30% reduction in crown density, the presence of 30% dead and/or drying branches in the upper half of the crown; the presence of damage (nutrition, burn, chlorosis, necrosis, etc.) and the exclusion of 30% of the leaf surface from assimilation activity.
− Level 3 (severely damaged (severely weakened) tree)—The presence of at least one of the following signs: decrease in crown closure by 60% due to premature leaf fall or thinning of the skeletal part of the crown, presence of 60% of dead and (or) drying branches in the upper half of the crown, damage by various factors and exclusion of 60% of the leaf surface from assimilating activity; presence of death of the upper part of the crown.
− Level 4 (drying tree)—The crown is destroyed, its closure is at least 15–20% of a healthy one; >70% of the branches, including the upper half, are dry or pale green, yellowish, orange-red in color, necrosis is whitish, brown or black, signs of pest damage are possible in the butt and middle parts of the trunk.
− Level 5 (fresh and old deadwood)—Dead trees. They may contain remains of dry needles or leaves, and the bark and small twigs are often intact.

They are typically infested with xylophagous insects. Microsoft^®^ Excel^®^ LTSC MSO (version 2505, Assembling 16.0.18827.20102) (Microsoft Corporation, Redmond, WA, USA) was used for statistical data processing.

## 4. Results and Discussion

### 4.1. Current State of Forest Vegetation and Tree and Shrub Communities Composition

Forest vegetation currently covers only 7.7% (204.1 thousand ha) of the study area, with over 70% comprising artificial plantations [10]. According to 2015 data [27], the forest fund consists of 60.4% forestry and game management areas, 27.0% agricultural sector (agroforestry complex, field-protection plantations), 4.2% road-transport network protective plantations, 1.0% water-protection plantations, and 2.3% other forests and plantations. The nature reserve fund currently occupies 111,033 ha, of which natural forest stands—represented by ravine, watershed, and floodplain forests—constitute merely 5.1%. The proportion of native species in artificial forest plantations reaches up to 80%, including 50% in field-protection belts [27], whereas in the green infrastructure of regional urban ecosystems, including the road-transport network, it does not exceed 35% [10,27].

The regional tree and shrub vegetation includes 135 species, of which the native fraction is 85 species (63%) from 34 genera and 17 families, and the adventitious fraction is 50 species (37%) from 38 genera and 23 families [47,48]. Fragmentation of natural tree and shrub communities in the steppe zone and intensifying anthropogenic transformation, which promotes the active spread of alien species in artificial plantations, lead to the adventization of the flora and vegetation, with extremely negative ecological consequences manifested in dysfunctional ecosystem performance and an unpredictable trajectory of further ecosystem development [8].

The overwhelming majority of adventive species are ergasiophytes, i.e., cultivated species that have escaped from cultivation, where they were originally introduced for landscaping and decorative, protective, or environment-forming functions. The widespread introduction of non-native species—not only within urban ecosystems but throughout the extensive network of field-protection and roadside belts and other types of forest plantations—accelerates the process of alien species invasion. Over a 10-year period alone (2010–2020), the proportion of adventive woody plant species in the regional tree and shrub communities increased by 5% (12 species) [48]. The prolonged taxonomic and quantitative dominance of introduced species in green plantations leads to their acclimatization and uncontrolled dispersal—initially along anthropogenically transformed territories and subsequently, with potential invasion into natural plant communities.

**Figure 2:**
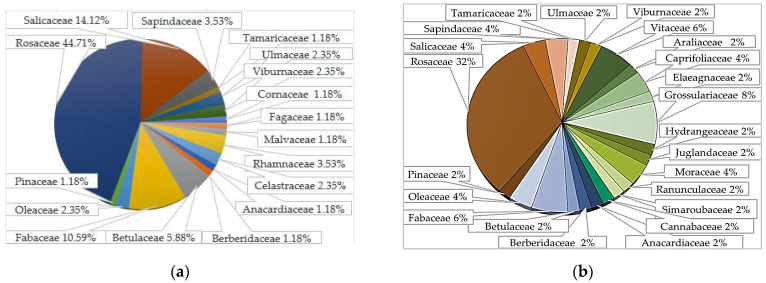
Taxonomic diversity of native (**a**) and adventive (**b**) woody plant species at the family level.

Most alien species are found primarily in anthropogenically disturbed ecotopes, within urban ecosystems, near transport routes, etc. It is noteworthy that 30 species of the adventitious flora belong to taxa phylogenetically close to native species. A comparison of the composition of the native and adventitious flora fractions revealed some similarities in the taxonomic spectrum at the family level (Figure 2). In particular, 60% of adventitious species are representatives of closely related and dominant taxa in the native flora. Among the eight shared families, the Rosaceae family is most widely represented in both fractions. This is explained by its high adaptive potential, making the family almost cosmopolitan. Representatives of this family adapt quite well not only to the challenging natural and climatic conditions of the region but also to the adverse factors of anthropogenic pollution characteristic of the study area. Relationships are also observed among lower taxonomic categories (subfamilies, tribes, genera).

Almost a third of native flora genera (11 out of 34) have representatives among alien species. This may indicate a genetic predisposition that increases the likelihood of invasive naturalization with unpredictable consequences. At the same time, it suggests that closely related native species, given their specific characteristics, can serve as alternatives to introduced species used in plantings for various purposes. Examples include the easily replaceable species of the genus *Acer* L. and *Populus* L.

### 4.2. Ecomorphological Characteristics of Native Tree and Shrub Communities

To assess the adaptive and functional potential of native species and the efficiency of their utilization in artificial plantations of various purposes, their ecomorphological, chorological, biomorphological, phytocoenotic, ontogenetic, and phenorhythmotypic characteristics were analyzed.

Natural tree–shrub vegetations of the southern East European Plain are confined predominantly to areas with dissected relief—wet gullies, ravines, hollows, and major river valleys [27]. The diversity of geomorphological, microclimatic, hydrological, and petrographic conditions generates a considerable mosaic of ecotopes within gullies. Forest vegetation is associated with more leached positions, linked to the upper reaches of gullies and predominantly north-facing slopes [37,38]. Notably, native woody and shrub species represent not only forest and shrub communities but also meadow, psammophytic, halophytic, steppe, and chasmophytic vegetation on granite outcrops, which significantly broadens the ecomorphic spectrum of the native tree and shrub communities. In studying the range of ecological characteristics of native species, we considered only the most important ones, which determine the viability and normal functioning of plantings. These include their relationship to light, moisture, fertility, acidity, salinity, and soil texture.

In the heliomorphic structure of the tree and shrub communities, the overwhelming majority are light-demanding species (83 species, 98%), of which 24 tolerate light or temporary shading, while two species are associated with shaded habitats but readily adapt to more intense illumination.

**Figure 3:**
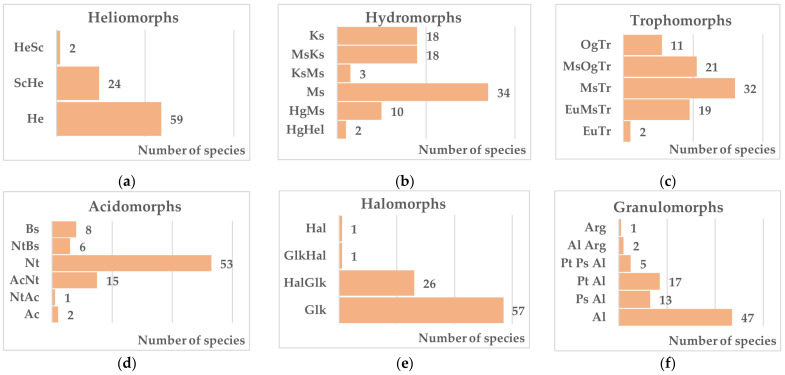
Ecomorphological structure of the native tree and shrub communities. Notes: (**a**): He—heliophytes, ScHe—scioheliophytes, HeSc—heliosciophytes; (**b**): Ks—xerophytes, MsKs—mesoxerophytes, KsMs—xeromesophytes, Ms—mesophytes, HgMs—hygromesophytes, Hg—hygrophytes, HgHel—hygrohelophytes; (**c**): EuTr—eutrophs, EuMsTr—eumesotrophs, MsTr—mesotrophs, MsOgTr—mesooligotrophs, OgTr—oligotrophs; (**d**): Ac—acidophiles, NtAc—neutroacidophiles, AcNt—acidoneutrophiles, Nt—neutrophiles, Bs—basiphiles; (**e**): Glk—glycophytes, HalGlk—haloglycophytes, GlkHal—glycohalophyte; (**f**): Pt—petrophytes, Ps—psammophytes, Al—alevritophytes, Arg—argilophytes.

A number of shrubs (18 species) participating in steppe and chasmophytic vegetation exhibit relatively high drought resistance. The xerophilic series is well represented in the native tree and shrub communities (Figure 3), supplemented by 18 mesoxerophytes. The greatest number of drought-resistant woody species belongs to the families Rosaceae Juss. (19 species) and Fabaceae Juss. (all nine regionally represented species). A characteristic feature of xerophilic species is their association with infertile rocky or sandy soils with neutral or alkaline reaction. The majority (17 species) tolerate temporary and/or slight soil salinity, while *Tamarix gracilis* Willd. is naturally associated specifically with saline substrates.

Drought and salt tolerance are key selection criteria for roadside plantings, as these traits best suit the following ecotopic conditions: elevated temperatures during the warm season due to heating of the road surface, constant air currents that increase aridity, and the use of de-icing agents in winter that salinize the soils. Overall, 28 species demonstrate varying degrees of evolutionary adaptation to saline soils (Figure 3). Some studies [31,49] provide evidence that plant tolerance to chloride-sulfate soil salinity and high cation exchange capacity are key indicators of gas tolerance. According to these authors, who studied regional tree–shrub vegetation species phylogenetically related to saline chloride-sulfate soils demonstrate the greatest gas tolerance, while acidophilic moisture-loving plants demonstrate less resistance to atmospheric pollution. Among the tree species evaluated in urban conditions, local species such as *Pyrus communis* L., *Cotinus coggygria* Scop., and *Quercus robur* L. were classified as the most resistant group, demonstrating the highest capacity for gas absorption under constant atmospheric pollution by SO_2_ [31]. Such indicators are especially important for regions with increased anthropogenic load.

In our previous research on biomorphological analysis and condition assessment of woody species in plantations along a main thoroughfare of the Donetsk–Makeyevka agglomeration [5], disturbance or absence of vertical stratification was revealed, substantially reducing protective functions. To enhance plantation functionality and reduce the burden on tall tree species performing primary protective and environment-forming functions, the proportion of low-growing trees and shrubs in linear plantings must be increased. Owing to their ecological characteristics, native shrubs and low-growing trees of the xerophilic series, exhibiting varying degrees of salt tolerance, represent a potentially valuable resource for protective roadside plantations—forming the lower and middle tiers—as well as for median strip landscaping.

Despite individual phylogenetically formed ecological characteristics, native species—with few exceptions—can be grouped into three ecomorphological categories according to their edaphic factor associations, broadly determining their relevance to specific structural elements of the ecological network (Table 1).

Considering the averaged individual ecomorphological characteristics and the inherent amplitude of tolerance exhibited by most species, the composition of the native tree and shrub communities, despite its limited species representation, is sufficient for establishing plantations across areas with diverse ecological conditions.

### 4.3. Chorological Analysis and Ecological Plasticity

**Figure 4:**
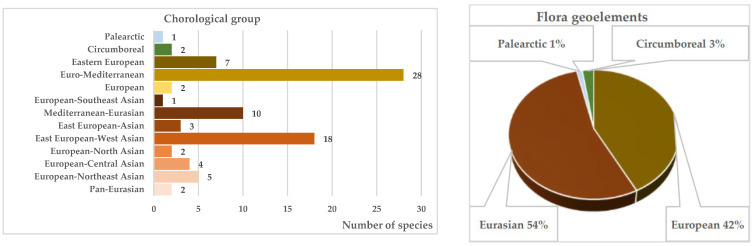
Chorological structure of the native fraction of the tree and shrub communities of the steppe zone of the southern East European Plain.

For an indirect assessment of ecological plasticity, the chorological spectrum of the native tree and shrub, species range sizes, the position of the study area within range structures, and climatic and natural zonality within ranges were examined. The identified chorological elements indicate a relatively complex genesis, influenced by the floras of various floristic subkingdoms. The chorological structure is represented in nearly equal proportions by European and Eurasian geographical elements (Figure 4). A minor proportion represents the broader Circumboreal and Palaearctic geo-elements. The European-Mediterranean (32%) and Eastern European–Western Asian (22%) chorological groups hold the leading positions (Figure 4), with their florogenesis associated with progressive xerophilization of climatic conditions. The dominance of these chorological groups is characteristic of the steppe zone flora of the southern East European Plain, indicating a high degree of adaptation to regional climatic conditions.

In our previous studies evaluating the viability of introduced woody species under roadside plantation conditions, the highest longevity and vitality indicators were predominantly found among Mediterranean flora representatives [5], reflecting the substantial influence of this flora on steppe zone florogenesis. This confirms the effectiveness of species selection for introduction based on florogenetic affinity [50] and demonstrates the advisability of maximizing native European-Mediterranean species in regional green infrastructure development.

**Figure 5:**
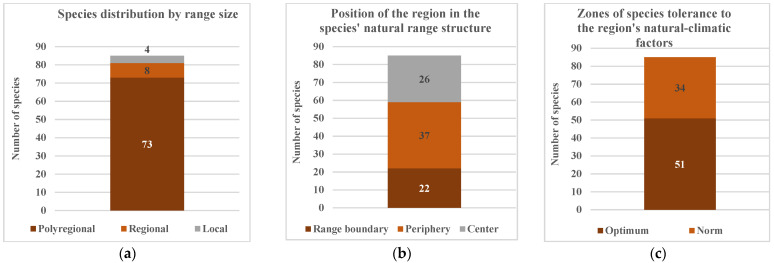
Ecological–chorological characteristics of the native fraction of the regional tree and shrub communities: (**a**) species distribution by range size; (**b**) position of the region in the species’ natural range structure; (**c**) zones of species tolerance to the region’s natural–climatic factors.

Species with extensive polyregional ranges predominate (86%) in the tree and shrub communities (Figure 5a), covering multiple floristic districts, likely reflecting a wide amplitude of ecological plasticity conditioned by significant adaptive potential of their gene pools. Species with local and regional ranges are mostly vulnerable, and nearly half (*Calophaca wolgarica* (L.f.) Pall. ex Fisch., *Caragana scythica* (Kom.) Pojark., *Crataegus ucrainica* Pojark., *Genista albida* Willd., *Tamarix gracilis* Willd.) possess conservation status. Their decline is primarily attributable to complete displacement from natural habitats, ubiquitous population fragmentation, and gene pool depletion resulting from total anthropogenic transformation territory of their distribution. Despite possessing economically valuable traits, most of these species are rarely used in cultivation due to their low resistance threshold, likely a result of their depleted gene pool. However, these species require special attention. Artificial propagation methods are essential. Increasing their populations and actively integrating them into green infrastructure, including ecological network areas, are currently the only methods for preserving their gene pool and regional biodiversity as a whole.

Within the structure of many ranges, the study area is situated at the periphery (44%) or at the distribution boundary (26%) (Figure 5b). This would suggest deteriorating living conditions; however, based on the correspondence of their ecomorphological structure to regional climatic conditions, many species are within the zone of optimum or norm (Figure 5c). This apparent contradiction can be explained by two factors. First, in the absence of insurmountable natural barriers at range peripheries, particularly when populations are fragmented and isolated, intensive form generation is initiated, leading under natural selection to increased acclimatization and adaptation. Second, a well-substantiated hypothesis posits secondary steppification of large areas within this territory, which, until the 16th–17th centuries, supported extensive forests [27,51]. Deforestation resulted from human activity rather than climate change; consequently, the current range boundaries of many woody species do not reflect their ecological dispersal potential.

Moreover, 90% of the study territory is presently anthropogenically transformed and generally inaccessible for natural dispersal of most plant and animal species, necessitating restoration and expansion of the ecological corridor network using native species to re-establish at least minimal natural biotic flow connecting populations and maintaining evolutionary processes.

**Figure 6:**
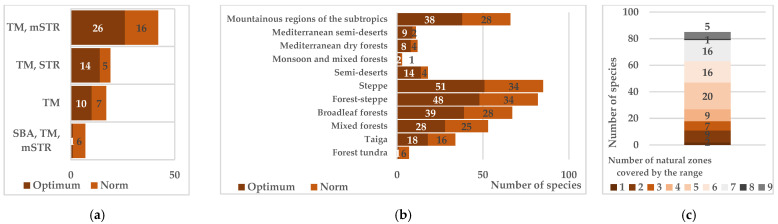
Distribution of species by their range coverage of climatic (**a**) and natural (**b**) zones and the level of polyzonality (**c**). Notes: SBA—subarctic zone, TM—temperate, STR—subtropical, mSTR—mountain regions of the subtropical zone.

The high ecological plasticity of most species is further confirmed by the fact that polyregional ranges encompass territories with different climatic and natural zones (Figure 6a,b). More than half the species also occur in mountainous regions of the temperate and subtropical zones, at elevations up to 3000–3500 m a.s.l. Despite its limited species composition, the native flora is unique in including species of the northern polyzonal group—cold-resistant plants whose range extends to the taiga (*Acer platanoides* L., *Euonymus verrucosus* Scop., *Frangula alnus* Mill., *Ulmus glabra* Huds., *Viburnum opulus* L., etc.) and forest–tundra (*Betula pendula* Roth, *Pinus sylvestris* L., *Populus tremula* L., *Prunus padus* L., etc.)—as well as the temperate polyzonal group—thermophilic and drought-resistant species capable of surviving Mediterranean semi-desert conditions (*Berberis vulgaris* L., *Cotinus coggygria* Scop., *Rosa spinosissima* L., *T. gracilis*). Up to 87% of the native tree and shrub communities’ species are polyzonal to varying degrees (Figure 6c).

Species with polyregional/polyzonal ranges, characterized by high ecological plasticity, adapt well to technogenic ecotope conditions. High tolerance has been documented in polyzonal species, including *Alnus glutinosa* (L.) Gaertn., *Cornus sanguinea* L., *Corylus avellana* L., *Malus sylvestris* (L.) Mill., *Pyrus communis* L., *Rosa canina* L., *Salix alba* L., and *Tilia cordata* Mill., employed alongside introduced species in phytoreclamation of industrial quarries and dumps in Donbass [27]. Self-regeneration has been observed in certain species (*Acer tataricum* L., *B. pendula*, *C. coggygria*, *P. tremula*, *Prunus spinosa* L., *Quercus robur* L.) within such plantations. All polyzonal species also possess extensive cultigenic ranges.

### 4.4. Phytocoenotic Affiliation and Community Modeling

**Figure 7:**
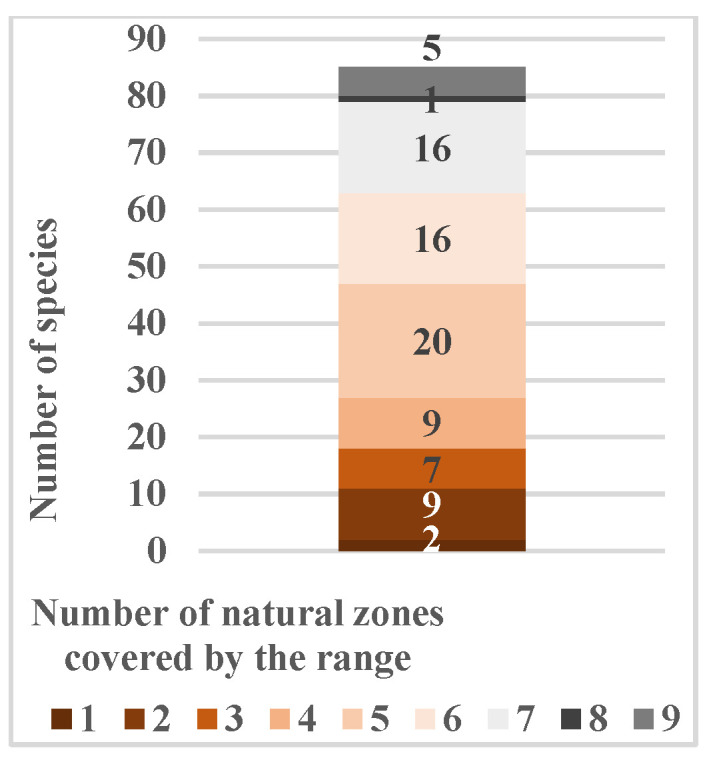
Native species represented in different vegetation types.

From the perspective of steppe zone characteristics, the overwhelming majority of native woody species represent intrazonal vegetation (ravine, floodplain, watershed, and cliff forests). Only 12 species participate in typical steppe vegetation (Figure 7), all belonging to two families—Rosaceae (eight species) and Fabaceae (four species)—which constitute the core of the xerophilic group. These are predominantly low-growing shrubs and subshrubs, occurring in various herb–grass communities of the classes *Festuco-Brometea* Br.-Bl. et Tx. ex Soó 1947, *Festucetea vaginatae* Soó ex Vicherek 1972, and on the Azov Sea coast, *Ammophilletea* Br.-Bl. et R. Tx. 1943.

**Figure 8:**
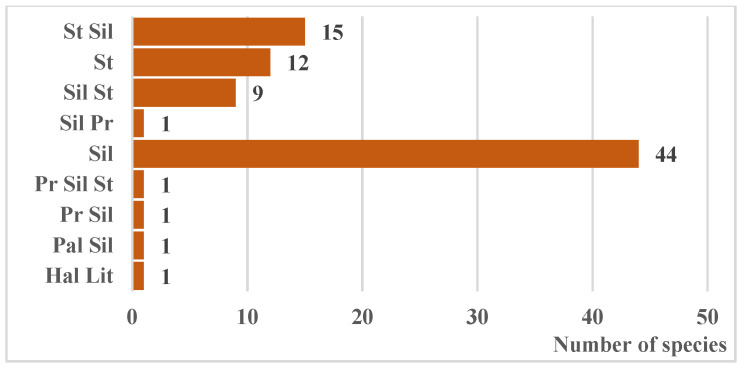
Ratio of coenomorphs in the native fraction of the regional tree and shrub communities. Notes: Sil—sylvants, St—stepants, Pr—pratants, Pal—paludants, Hal—halophytes, Ps—psammophytes, Ptr—petrophytes.

Analysis of phytocoenotic affiliation revealed that native species participate in at least 105 associations of various vegetation types (forest, shrub, meadow, steppe, psammophytic, chasmophytic) [39]. In addition to the principal coenomorphs (steppe and forest), 34% of species belong to transitional forms (Figure 8), significantly broadening the spectrum of their potential utilization in forming artificial florocomplexes at various green infrastructure or ecological network sites.

Associations in which the studied species occur can serve as models for creating artificial communities approximating natural ones. Their floristic composition typically includes decorative herbaceous native species suitable for the lower tier of plantings. In associations of any vegetation type, complexes of evolutionarily co-adapted arboreal and herbaceous species can be identified for modeling artificial communities, as in the following steppe vegetation associations:

*Veronico austriacae-Chamaecytisetum austriaci* Korotchenko et Didukh 1997—*Caragana frutex* (L.) K. Koch, *Chamaecytisus austriacus* (L.) Link, *Astragalus dasyanthus* Pall., *Phlomis pungens* Willd., *Veronica austriaca* L.;

*Vinco herbaceae-Caraganetum fruticis* Korotchenko et Didukh 1997—*C. frutex*, *Ch. austriacus*, *Prunus tenella* Batsch, *Adonis vernalis* L., *Asparagus officinalis* L., *Carex praecox* Schreb., *Melica transsilvanica* Schur, *Vinca herbacea* Waldst. & Kit., *Vincetoxicum hirundinaria* Medik.);

Shrub vegetation:

*Euonymo-Prunetum stepposae* Fitsailo 2006—*Euonymus europaeus* L., *P. spinosa*, *P. tenella*, *Rhamnus cathartica* L., *Coronilla varia* L., *V. herbacea*, *Viola ambigua* Waldst. & Kit.;

*Roso-Juniperetum* Tx. 1974—*Ligustrum vulgare* L., *Rosa × andegavensis* Bastard, *R. rubiginosa* L., *Fragaria viridis* Duchesne, *Salvia verticillata* L., *Teucrium chamaedrys* L.;

Forest vegetation:

*Aceri platanoidis-Fraxinetum excelsioris* Onyshchenko 1998—*Acer campestre* L., *A. platanoides* L., *Carpinus betulus* L., *Corylus avellana* L., *E. europaeus*, *E. verrucosus*, *Sambucus nigra* L., *U. glabra*, *Anemonoides ranunculoides* (L.) Holub, *Asarum europaeum* L., *Convallaria majalis* L., *Corydalis cava* (L.) Schweigg. et Koerte., *C. solida* (L.) Clairv., *Gagea lutea* (L.) Ker Gawl., *Galanthus nivalis* L., *Galium odoratum* (L.) Scop., *Geum urbanum* L., *Lamium galeobdolon* (L.) L., *L. maculatum* L., *Polygonatum multiflorum* (L.) All., *Pulmonaria obscura* Dumort., *Scutellaria altissima* L., *Stellaria holostea* L.;

*Stellario holosteae-Aceretum platanoidis* Bayrak 1996—*A. campestre*, *Fraxinus excelsior* L., *E. europaeus*, *Quercus robur* L., *T. cordata*, *Aegopodium podagraria* L., *A. ranunculoides*, *A. europaeum*, *C. solida*, *Ficaria verna* L., *L. maculatum*, *Lathyrus vernus* (L.) Bernh., *P. multiflorum*, *P. obscura*, *Scilla siberica* Haw., *Tulipa sylvestris* ssp. *australis* (Link) Pamp.

As a basic component for forming the lower tier in artificial tree and shrub plantations, native species of the families Cyperaceae Juss. and Poaceae Barnhart may also be used, whose resource potential has been examined in our earlier work [34,52]. For example, for shaded areas with arid conditions, the herbaceous tier base may consist of *Bromus inermis* Leyss., *C. humilis*, while in the moister ecotopes it may consist of *Milium effusum* L., *C. digitata* L., *C. elata*, which are quite widely represented in various natural plant communities.

Choosing a variety of plant species for tree and shrub plantations with different functionalities, taking into account the coevolutionary relationships of plants, reduces the risk of competitive interactions and promotes the sustainability of artificial communities. On the one hand, the phytocoenotic approach allows for the imitation of natural landscapes, which have become quite popular in recent decades from an esthetic point of view. On the other hand, plantings created using such principles have a significant environmental impact. This is especially effective for network structures such as natural forests, shelterbelts, and the like.

### 4.5. Biomorphological Characteristics and Ornamental Potential

In addition to high adaptive capacity and plant compatibility, habitual characteristics and individual development features are of considerable importance when designing tree and shrub plantations for any green infrastructure or ecological network component. The most significant parameters for planning plantation structure and composition were examined: biomorphological composition, plant height, growth rate, and ornamental value.

**Figure 9:**
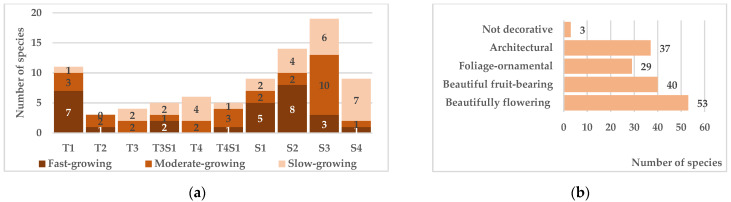
Plant groups by biomorph, height, and growth rate (**a**), and representation by ornamental traits (**b**).

According to the generally accepted classification [53], the native tree and shrub communities exhibit the following biomorphological structure: megaphanerophytes (14 species), mesophanerophytes (15), microphanerophytes (26), and nanophanerophytes (30). According to the classification by (Sokolov, 1965), which is more practical for use in crop production, the flora includes four groups each of trees and shrubs and two groups (T3S1, T4S1), combining transitional forms (Figure 9a). The distribution demonstrates that shrubs and low-growing trees dominate, determined by regional climatic conditions. For urban green infrastructure, however, fast-growing trees of the first-second magnitude (T1–T2) are preferred, performing environment-forming and protective functions more effectively due to greater biomass. The use of introduced species for urban greening is thus justified; however, the current balance of fast-growing tall plants—approximately 7:1 in species composition and 3:1 in numbers [5]—should be revised in favor of native species. For other facilities (field-protection, water-protection, forest amelioration, roadside plantations), the existing biomorphological diversity of the native flora is fully sufficient.

Regarding growth rate, the overall species composition is evenly distributed, but fast-growing species dominate among tall trees and shrubs, partly explaining their prevalence in urban landscaping. Studies demonstrate that slow-growing species possess advantages in other parameters—greater resistance to mechanical loads (both static and kinetic) and, under elevated anthropogenic pressure, higher viability and longevity [19]. The sufficient representation of native species across growth rate categories provides an opportunity to select appropriate assortments considering the planned specificity of green plantation functioning.

The species composition is also fairly representative in ornamental characteristics (Figure 9b), including ornamental flowering, ornamental-fruiting, and ornamental-foliage species, as well as architectural plants with compact, regularly shaped crowns. Only three species of *Rubus* L. lack ornamental value and are therefore suitable only as a lower tier in forest amelioration and water-protection plantations. This diversity is sufficient for the formation of plantings whose primary functions are habitat-forming and nature conservation. The use of highly ornamental introduced species for recreational areas and other esthetically pleasing sites remains a priority.

**Figure 10:**
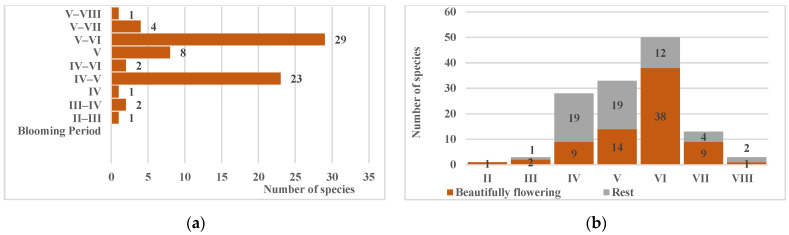
Groups by flowering period (**a**) and probabilistic distribution of flowering species throughout the growing season (**b**).

Although mass flowering of woody species occurs in May–June, flowering of individual species of the native tree and shrub communities can be observed from February to August (Figure 10). Depending on meteorological conditions, flowering phenophases may shift in either direction by 2–3 weeks to a month, but overall, from April to July, the simultaneous flowering of 1 to 20–30 species can be observed. Large-flowered species are absent among them, but due to the large inflorescences of species such as *Crataegus pentagyna* Waldst. & Kit. ex Willd., *Sambucus nigra* L., *Viburnum opulus* L. and/or the profuse flowering of *Caragana frutex* (L.) K. Koch, *Pyrus pyraster* (L.) Burgsd., *Spiraea hypericifolia* L., etc., a significant ornamental effect is achieved. Furthermore, ornamental entomophilous plants attract a large number of pollinating insects, including representatives of the local entomofauna, thereby preserving their ecological niche and contributing to the normal existence and reproduction of local populations, which is critically important for maintaining regional biodiversity and ecosystem resilience.

**Figure 11:**
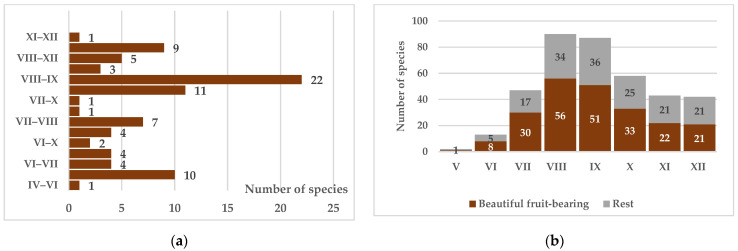
Groups by fruiting period (**a**) and probabilistic distribution of species with fruits, including those with mature but non-abscised fruits (**b**).

The number of species with ornamental fruits is somewhat lower, with fruit ripening occurring from April to October (Figure 11a). In many species, fruits are retained without falling even during winter months (Figure 11b); the predominantly red coloration contrasting with green foliage supplements the ornamental effect created by flowering and provides accent elements during the leafless period. All fruits and seeds are readily consumed by birds and serve as initial links in food chains for numerous faunal representatives.

The use of two shrub species should be restricted to areas distant from agricultural lands. *Berberis vulgaris* L. serves as an alternate host for *Puccinia graminis* Pers. f. sp. *tritici*, causing stem rust in cereals. *Rhamnus cathartica* L. is the primary host for overwintering eggs of soybean aphid (*Aphis glycines* Matsumura) and an alternate host for oat crown rust (*Puccinia coronata* Corda).

Based on the comprehensive analysis of individual traits determining adaptive capacity, ecological affiliation, and potential functional load, all native tree and shrub communities species were assigned to utilization groups for various plantation types (Application. Table 2). Species representation across groups permits optimal assortment selection for individual sites, considering specific ecotope conditions and planned functionality. Approximately 29 species are universal and can serve as a foundation in any plantation type.

### 4.6. Native Species in Roadside Landscaping

To determine the effectiveness of using native species in the green infrastructure of industrial cities and their resilience to anthropogenic impacts, a comparative analysis of the status of native and introduced species in roadside plantings within the Donetsk–Makiivka agglomeration was conducted.

Our analysis of the taxonomic composition and condition of roadside trees and shrubs along a section of one of the main highways of the Donetsk–Makiivka agglomeration revealed that introduced species [5] dominate in species composition (78 species from 48 genera and 25 families—78%) and abundance (5509 specimens—73%) [5].

**Figure 12:**
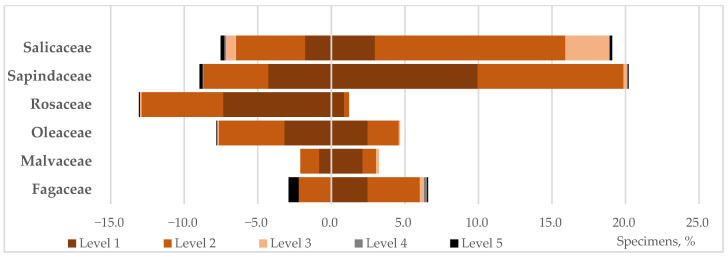
Representation by family and condition of trees and shrubs in roadside plantings: introduced species on the left, native species on the right.

The native flora in the plantings is represented by 23 species from 16 genera and 12 families (2670 specimens). This represents only 27% of the recommended number of species, indicating that the potential for landscaping of species phylogenetically adapted to the natural and climatic conditions of the region is far from exhausted. In terms of species representation, the Rosaceae family (22 species) dominates among the introduced species. Notably, among the native trees and shrubs, this family has the highest species richness (38 species), accounting for half of the species composition of the native flora. However, no more than 10 species are used in urban landscaping, typically represented by small groups or single specimens. Only four species were used in roadside landscaping in the study area. To compare viability levels, only taxonomically related native and introduced species were considered (Figure 12).

Although the results obtained cannot be considered statistically reliable due to the insufficient number of some species in the sample, a certain trend is still evident. The percentage of healthy plants in the sample by family (introduced/native): Salicaceae—24/15, Fagaceae—none/38, Malvaceae—40/87, Oleaceae—41/53, Rosaceae—56/72, Sapindaceae—49/49.

**Figure 13:**
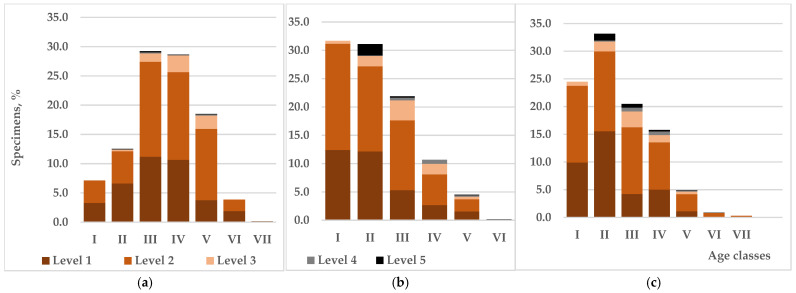
Levels of viability of tree species in different age classes: I—up to 10 years; II—11–20; III—21–30; IV—31–40; V—41–50; VI—51–60; VII—61–70: (**a**)—native species, (**b**)—taxonomically close introduced species, (**c**)—introduced species in general.

According to the results obtained, native species are comparable to introduced species in terms of viability under conditions of increased anthropogenic stress, with the exception of representatives of the Salicaceae family. This is due to the fact that among the native species of this family, *Populus alba* L. is predominantly represented (860 of 922 specimens). This species belongs to the group of tall trees (>25 m), has a pyramidal, compact crown, and is highly resistant to mechanical stress, including wind. Like other poplar species, it has a significant leaf mass and plays a key role in creating a favorable microclimate for city residents. Among poplars, this species is one of the most beautiful, as well as the most resistant to drought and frost. However, according to our observations, in linear roadside plantings in the immediate vicinity of highways, viability significantly decreases after plants reach 45–50 years of age. Based on long-term observations, we have shown that this age is critical in roadside landscaping for many introduced species as well [5,11,19]. In the plantings we studied, 42% of *P. alba* specimens reached the critical age, and a decrease in viability was predominantly observed in this group. The overwhelming majority of introduced species simply do not survive to this age (Figure 13). In general, the age structure of woody plants illustrates that, despite a comparable degree of suppression under conditions of increased anthropogenic pressure, native species have a longer lifespan than introduced species, including closely related ones. The age threshold for declining viability in roadside plantings also differs. Thus, among native species, a sharp deterioration in condition is observed after 40 years, while among introduced species, this deterioration occurs after 30 years, both for closely related and many other species (Figure 13).

Along with poplars, species of the genus *Acer* L. (Sapindaceae) also form the basis of the roadside landscaping of the study area. Both introduced and native species of this genus have a similar level of adaptation to anthropogenic loads. Both are well represented in the roadside landscaping of the study area: natives—three species, 973 individuals; introduced species—three species, 748 individuals. The advantage of the introduced species (*A. pseudoplatanus* L., *A. saccarinum* L. and *A. negundo* L.) is that they belong to the group of fast-growing tall species preferred for roadside landscaping. However, one of them, *A. negundo*, is a rather aggressive invasive species; its cultivation in this area is prohibited by law, and it is gradually being removed from the plantings. All native species (*A. campestre* L., *A. platanoides* L., *A. tataricum* L.) are moderately growing and shorter, but in combination with tall and fast-growing native species of other genera and families (*P. alba*, *Ulmus glabra* Huds., *U. laevis* Pall., *Betula pendula* Roth, etc.), they can completely replace introduced species in roadside plantings that serve as ecocorridors. Given the status of native species growing under conditions of increased anthropogenic pressure, it is advisable to recommend their wider use in the formation of green infrastructure in urban ecosystems.

## 5. Conclusions

The study area is characterized by large-scale anthropogenic transformation and critical fragmentation of natural biotopes. The conservation and self-healing of the remaining natural ecosystems can be facilitated by restoring damaged ones and creating new ecological corridors that reliably facilitate interactions between them. Such corridors can be supported by a comprehensive network of tree and shrub plantings that perform various functions (shelterbelts, roadside and water-protecting plantings, green infrastructure of urban ecosystems, etc.). Highly effective biological flows that ensure the conservation of regional biodiversity are possible only with the use of native plant species in such plantings. The results of the conducted research show that, based on the characteristics that determine their functional value, native tree and shrub species can completely replace introduced species in various types of plantings outside urban ecosystems. When creating green infrastructure within urban ecosystems, complete replacement of introduced species is impossible due to the lack of certain categories of functionally valuable traits in native species. However, given their high viability and effectiveness in plantings that meet their functional capabilities, as well as their additional conservation function, it is advisable to increase their share in plantings, which currently stands at 3:7, in favor of introduced species, both in species composition and quantity. The predominance of native species should be primarily ensured in roadside plantings, which effectively serve as a means of biological flow.

## Figures and Tables

**Table 1:** Options for utilizing species of different edaphic groups in anthropogenic ecotopes of the regional ecological network.

Edaphic Eco-Groups	Share, %	Characteristics	Correspondence to Ecological Network Elements
Eu-,MsTr-Ac,Nt-Glk-Al	29	fertile, nutrient-rich, neutral or slightly acidic, non-saline, sandy loam or loamy	field-protection belts, water-protection and artificial forest plantations, recreational zones of urban ecosystems
MsTr-Nt-Glk-Al	30	moderately fertile, neutral, non-saline, loamy/sandy loam	forest amelioration plantations, residential zones of urban ecosystems
Ms-, OgTr-Nt, Bs-HalGlk- Ps,Pt, Al	41	infertile, slightly alkaline (alkaline) or neutral, solonetzic, rocky and/or sandy, sandy loam	road-transport network, industrial zones, technogenically altered territories (spoil heaps, dumps, quarries, etc.)

**Table 2:** Habitual traits, features of individual and seasonal development of native tree and shrub communities’ species, and their utilization groups in various plantation types.

Species	Flowering/Fruiting Periods	Groups			Groups by Usage				
		Biomorph and Height	Growth Rate	Ornamental Value	1	2	3	4	5
*Cotinus coggygria*	V–VI/VIII	T4, S1	Mg	FL, LF	+	+	+	+	+
*Berberis vulgaris*	IV–V/IX–X	S2	Fg	FL, FR, LF	–	–	–	–	+
*Alnus glutinosa*	IV–V/X	T2	Fg	LF, AP	–	–	–	+	+
*Betula pendula*	IV–V/VIII	T1	Fg	AP	–	–	+	+	+
*B. pubescens*	V/IX	T3	Sg	AP	–	–	–	+	+
*Carpinus betulus*	IV–V/IX–X	T4	Sg	LF, AP	+	+	+	+	+
*Corylus avellana*	II–III/VIII–IX	T4, S1	Sg	FL, LF, AP	+	+	+	+	+
*Euonymus europaeus*	IV–VI/VI–X	S1	Sg	FR, LF	–	–	+	+	+
*E. verrucosus*	V–VI/VIII–IX	S2	Sg	FR, AP	+	–	+	+	+
*Cornus sanguinea*	V–VI/VIII–IX	S1	Sg	FL, FR	+	+	+	+	+
*Calophaca wolgarica*	VI–VII/VI–VIII	S4	Sg	FL, LF	–	–	–	–	+
*Caragana frutex*	V–VI/VI–VIII	S3	Sg	FL	–	–	+	+	+
*C. frutex ssp. mollis*	V–VI/VI–VIII	S3	Sg	FL	–	–	–	+	+
*C. scythica*	IV–V/VI–VIII	S4	Sg	FL	–	–	–	–	+
*Chamaecytisus austriacus*	VI–VIII/IX	S4	Sg	FL,	–	–	–	–	+
*Ch. borysthenicus*	V/VII–VIII	S3	Sg	FL	–	–	–	–	+
*Ch. lindemannii*	V–VI/VII–VIII	S3	Sg	FL	+	+	–	–	+
*Genista albida*	IV–VI/VI–VII	S4	Sg	FL	–	–	–	–	+
*G. tinctoria*	VI–VII/VIII–IX	S3	Sg	FL	–	–	–	–	+
*Quercus robur*	IV–V/IX–X	T1	Sg	LF, AP	+	+	+	+	+
*Tilia cordata*	VI–VII/VIII–IX	T1	Mg	FL, AP	+	+	+	+	+
*Fraxinus excelsior*	IV–V/VIII–X	T1	Mg	AP	+	+	+	+	+
*Ligustrum vulgare*	VI/VII–XII	S2	Sg	FL, FR	+	+	+	+	+
*Pinus sylvestris*	-	Д1	Mg	LF, AP	–	+	–	+	+
*Frangula alnus*	V–VI/VIII–IX	S1, T4	Mg	FR, LF	–	+	–	+	+
*Rhamnus cathartica*	V–VI/VIII–IX	S1	Fg	FR	–	–	+	+	+
*Rh. saxatilis ssp. tinctoria*	V–VI/VIII	S1	Fg	FR, AP	–	+	+	–	+
*Cotoneaster laxiflorus*	V–VI/VIII–IX	S2	Sg	FR, AP	–	+	–	+	+
*Crataegus× kyrtostyla*	VI/IX	S1	Mg	FL, FR, LF	+	–	+	+	+
*C. ambigua*	V/VIII–IX	T4, S1	Mg	FL, FR, LF	+	+	-	+	+
*C. pentagyna*	V–VI/VIII–IX	T4	Sg	FL, FR, LF, AP	+	+	+	+	+
*C. monogyna*	V–VI/VIII–IX	T4	Sg	FL, FR, LF	+	+	+	+	+
*C. rhipidophylla*	V–VI/VI–X	T4	Mg	FL, FR, LF	+	+	+	+	+
*C. ucrainica.*	VI/X	T4	Sg	FL, FR, LF,	–	–	–	+	+
*Malus sylvestris*	V–VI/VIII–IX	T4, S1	Mg	FL, FR, LF, AP	+	+	+	+	+
*Prunus fruticosa*	III–IV/V	T4	Mg	FL, FR, AP	–	+	–	–	+
*P. padus*	V–VI/VIII–IX	T3	Mg	FL, FR, AP	–	–	+	+	+
*P. spinosa*	IV–V/VII–VIII	T4	Mg	FL, FR, AP	+	+	+	+	+
*P. tenella*	IV–V/VII–VIII	S3	Sg	FL	–	+	–	–	+
*Pyrus pyraster*	IV–V/VIII–IX	T3	Sg	FL, AP	+	–	+	+	+
*P. communis*	IV–V/VIII–IX	T3	Mg	FL, AP	+	–	+	+	+
*Rosa andegavensis*	VI/VII–XII	S2	Mg	FL, FR	–	–	+	+	+
*R. balsamica*	V/VI–XII	S3	Mg	FL, FR	+	–	+	+	+
*R. caesia*	VI/VII–XII	S3	Mg	FL, FR	+	–	+	+	+
*R. canina*	V–VI/VIII–XII	S2	Mg	FL, FR	+	–	+	+	+
*R. corymbifera*	V–VII/VII–XII	S2	Mg	FL, FR	–	–	+	+	+
*R. diplodonta*	V–VI/VII–XII	S4	Mg	FL, FR	–	–	+	+	+
*R. donetzica*	V–VI/VII–XII	S4	Mg	FL, FR	–	–	+	+	+
*R. dumalis*	VI–VII/VIII–XII	S3	Mg	FL, FR	+	–	+	+	+
*R. glabrifolia*	V–VI/VIII–XII	S3	Mg	FL, FR	+	–	+	+	+
*R. gorenkensis*	V–VI/VII–XII	S2	Mg	FL, FR	–	–	+	–	+
*R. livescens*	V/VI–XII	S4	Mg	FL, FR	+	–	+	+	+
*R. cinnamomea*	V–VII/VIII–XII	S3	Mg	FL, FR	+	–	+	+	+
*R. × malmundariensis*	V/VI–XII	S2	Mg	FL, FR	+	–	+	+	+
*R. micrantha*	VI/VII–XII	S3	Mg	FL, FR	+	+	+	+	+
*R. rubiginosa*	VI–VII/VIII–XII	S2	Mg	FL, FR	+	–	+	+	+
*R. spinosissima*	V–VI/VII–XII	S3	Mg	FL, FR	–	–	+	+	+
*R. × burgalensis*	V/VI–XII	S3	Mg	FL, FR	+	–	+	+	+
*R. tomentosa*	V–VI/VII–XII	S2	Mg	FL, FR	–	–	+	+	+
*R. villosa*	VI/VII–XII	S3	Mg	FL, FR	+	–	+	+	+
*Rubus caesius*	V–VIII/VII–IX	S3	Mg	-	–	–	+	+	–
*R. idaeus*	VI–VII/VII–VIII	S2	Mg	-	–	–	+	+	–
*R. saxatilis*	V–VI/VII–VIII	S4	Mg	-	–	–	+	+	–
*Spiraea crenata*	V–VI/VII	S3	Mg	FL, AP	+	+	+	+	+
*S. hypericifolia*	V–VI/VIII	S3	Mg	FL, LF	+	+	+	+	+
*Populus alba* L.	IV–V/VI–VII	T1	Fg	LF, AP	+	+	+	+	+
*Populus nigra* L.	IV–V/V–VI	T1	Fg	AP	+	+	+	+	+
*Populus tremula* L.	IV–V/VI–VII	T1	Fg	AP	+	+	+	+	+
*Salix acutifolia Willd.*	III–IV/V–VI	T3S1	Fg	AP	–	+	+	+	+
*Salix alba* L.	IV–V/V–VI	T1	Fg	AP	–	+	+	+	+
*Salix aurita* L.	IV–V/V–VI	S3	Fg	AP	+	+	+	+	+
*Salix caprea* L.	IV–V/V–VI	T3S1	Fg	FL, LF, AP	–	+	–	+	+
*Salix cinerea* L.	IV–V/V–VI	S1	Fg	LF, AP	+	+	+	+	+
*Salix pentandra* L.	V–VII/VII–X	T3S1	Sg	LF, AP	–	+	+	+	+
*Salix rosmarinifolia* L.	V/VI–VII	S4	Fg	LF, AP	+	+	–	+	+
*Salix triandra* L.	IV–V, VII–IX/V–VI	T4S1	Fg	AP	–	+	+	+	+
*Salix vinogradovii*	IV/V–VI	S1	Fg	LF, AP	+	+	+	+	+
*Acer campestre*	IV–V/IX–X	T2	Mg	AP	+	+	+	+	+
*A. platanoides*	IV–V/IX–X	T2	Mg	AP	+	+	+	+	+
*A. tataricum*	V–VI/IX–X	T4, S1	Sg	AP	+	+	+	+	+
*Tamarix gracilis*	V–VII/VIII	S2	Sg	FL, LF	–	+	–	–	+
*Ulmus glabra Huds.*	IV–V/V–VI	T1	Fg	LF, AP	+	+	+	+	+
*Ulmus laevis Pall.*	IV–V/V–VI	T1	Fg	LF, AP	+	+	+	+	+
*Sambucus nigra*	V–VI/VIII–X	S1	Fg	FL, FR, LF	+	+	+	+	+
*Viburnum opulus*	V–VI/VIII–X	S	Mg	FL, FR, LF	+	+	+	+	+
Total:					46	44	64	72	82

Note: Flowering/fruiting periods—indicate the time periods during which the phenophases of flowering and fruiting vary in the study area. Biomorph and height: T1—trees—first magnitude (>25 m), T2—second (15–25 m), T3—third (10–15 m), T4—fourth (<10 m); S1—shrubs—first magnitude (>3 m), S2—second (2–3 m), S3—third (1–2 m), S4—fourth (<1 m). Growth rate groups: Fg—fast-growing (mean annual increment ≥1 m), Mg—moderate-growing (0.5–0.6 m), Sg—slow-growing (≤0.25–0.3 m). Ornamental groups: Fl—ornamental-flowering, Fr—ornamental-fruiting, Lf—ornamental-foliage, Ap—architectural plants. Plantation type: 1—roadside; 2—forest amelioration (gully stabilization); 3—field-protection; 4—water-protection; 5—urban green infrastructure in recreational and residential zones; “+”—recommended for use, “–”—not recommended.

## Data Availability

The data presented in this study are available in the article.

## Abbreviations

The following abbreviations are used in this manuscript:

T1	Trees of the First Magnitude (>25 m)
T2	Trees of the Second Magnitude (15–25 m)
T3	Trees of the Third Magnitude (10–15 m)
T4	Trees of the Fourth Magnitude (<10 m)
S1	Shrubs of the First Magnitude (>3 m)
S2	Shrubs of the Second Magnitude (2–3 m)
S3	Shrubs of the Third Magnitude (1–2 m)
S4	Shrubs of the Fourth Magnitude (<1 m)
Fg	Fast-Growing
Mg	Moderate-Growing
Sg	Slow-Growing
FL	Ornamental-Flowering
FR	Ornamental-Fruiting
LF	Ornamental-Foliage
AP	Architectural Plants
He	Heliophytes
ScHe	Scioheliophytes
HeSc	Heliosciophytes
Ks	Xerophytes
MsKs	Mesoxerophytes
KsMs	Xeromesophytes
Ms	Mesophytes
HgMs	Hygromesophytes
Hg	Hygrophytes
HgHel	Hygrohelophytes
EuTr	Eutrophs
EuMsTr	Eumesotrophs
MsTr	Mesotrophs
MsOgTr	Mesooligotrophs
OgTr	Oligotrophs
Ac	Acidophiles
NtAc	Neutroacidophiles
AcNt	Acidoneutrophiles
Nt	Neutrophiles
Bs	Basiphiles
Glk	Glycophytes
HalGlk	Haloglycophytes
GlkHal	Glycohalophyte
Pt	Petrophytes
Ps	Psammophytes
Al	Alevritophytes
Arg	Argilophytes
Sil	Sylvants
St	Stepants
Pr	Pratants
Pal	Paludants
Hal	Halophytes
Ptr	Petrophytes
SBA	Subarctic Zone
TM	Temperate Zone
STR	Subtropical Zone
mSTR	Mountain Regions of the Subtropical Zone
